# Prevalence and Treatment Needs for Early Childhood Caries Among 3–5-Year-Old Children From a Rural Community in Uganda

**DOI:** 10.3389/fpubh.2019.00259

**Published:** 2019-09-18

**Authors:** Norman Musinguzi, Arthur Kemoli, Isaac Okullo

**Affiliations:** ^1^Department of Dentistry, School of Health Sciences, Makerere University, Kampala, Uganda; ^2^Department of Paediatric Dentistry and Orthodontics, School of Dental Sciences, University of Nairobi, Nairobi, Kenya

**Keywords:** early childhood caries, treatment needs, primary school children, Rukungiri District, Uganda

## Abstract

**Introduction:** Early Childhood caries (ECC) is the term used to describe dental caries in children aged 71 months and below. ECC causes a lot of pain and discomfort in the affected children and is expensive to treat. There is limited data on the prevalence of ECC in preschool children resident in the rural Uganda.

**Aim:** To determine the prevalence and treatment needs for ECC among 3–5-year-old nursery school children in a rural community in Rukungiri District, Uganda.

**Study Design:** This was a descriptive cross-sectional study in which 432 nursery school children aged 3–5 years from rural Nyakagyeme sub-county in Ugandan, participated. Informed consent for participation in the study by the children was obtained from their parents/guardians prior to the commencement of the study. The examination of the participants was done under field conditions, with the child lying his or her back of the neck on the PI's lap, with both of them seated on a bench and using natural light augmented with a headlamp to examine the oral cavity. The findings of the examination were recorded on individualized modified WHO Oral Health Assessment Forms for children.

**Data Analysis:** The data gathered were analyzed using Windows SPSS Version 23.0 computer programme, and descriptive results for the variables obtained and Mann Whitney and Kruskal–Wallis tests used to compare and relate the variables. The P<0.05 was considered statistically significant with the Confidence interval set at 95%. The findings were presented in Tables and Figures.

**Results:** A total of 230 (53.2%) male and 202 (46.6%) female participants, with 118 (27.3%), 145 (33.6%), 169 (39.1%) aged 3, 4, and 5 years old respectively, participated in the study. The overall prevalence of dental caries among the participants was 48.6%, with 11.6%, 18.5% and 18.5% recorded for the 3-, 4-, and 5-year old children, respectively. The male participants had a higher prevalence (26.1%) than the female counterparts (22.5%). The mean “**dmft**” for the participants was 2.04 (*SD* = 3.01) with the decay component (dt) having the highest value (*M* = 1.97, *SD* = 2.89), and contributing to 88.6% of the dental caries experience. The mean “**dmft**” was 1.79, 2.37 and 1.91 for the 3-, 4-, and 5-year old children, respectively, and the difference in the mean “**dmft**” among the age groups was not statistically significant.

**Conclusion:** The prevalence of ECC of 48.6% was moderate in this study population. The high level of unmet treatment need as exemplified by the high dental caries experience, was a cause for concern as this forms a risk factor for caries in the permanent dentition.

## Introduction

Dental caries remains a major public health burden for babies, pre-school, primary school going children and adolescents in both the developed and less developed countries (1, 2), with the greatest burden being borne by children from the disadvantaged communities (2). Early Childhood caries (ECC) is the term used to describe dental caries in children aged 71 months and below (3). The disease has a multifactorial etiology involving interaction of factors that include, a susceptible host tooth, micro-organisms with cariogenic potential S. mutans, S. sobrinus, and Lactobacili), a substrate (dental plaque and/or biofilm) that is suitable for the survival of the pathogenic flora and time for changes to take place (3–8). Diet is a critical factor in the etiology of dental caries and children who consume cariogenic carbohydrates frequently for longer periods have increased risk of developing ECC (2, 3, 9). Additionally, environmental factors like low socio-economic status, lower level of education and non-exposure to fluoride from the water supply and the other fluoride sources play a part in the process (3, 4, 6, 8, 9).

ECC affects the children's teeth with a distinctive pattern, with the smooth surfaces of the primary upper incisors being affected first, followed by the occlusal surfaces of the primary molars in succession as they erupt. The lower primary incisors are usually spared because of the protection provided by the tongue and the copious saliva from adjacent submandibular and sublingual glands (2, 5, 7, 8). Initially, the disease presents as a white chalk lesion of demineralised enamel, and if not arrested at this stage it will progress to enamel surface cavitation (5, 8). The rough cavities formed on the enamel surfaces become retentive areas for further accumulation of the dental plaque that potentiates the possible replication of the process leading to eventual total destruction of the tooth (8).

ECC causes a lot of discomfort besides pain and loss of the affected tooth. ECC can also affect feeding, speech development, and psycho-social well-being of the child (6), not to mention expensive treatment in the form of restorations, pulpotomies/pulpectomies, extractions, and space management than is often needed. In cases where the child is uncooperative and basic behavior modification techniques have failed, conscious sedation or general anesthesia may have to be used, and these are expensive management modes (2).

The reported prevalence of ECC from the few studies conducted in Uganda has ranged from 17.6 to 65%. However, these studies have been conducted mostly in the urban and peri-urban communities (10–12). Although these reported prevalence rates appear to be high, they are still few studies and Uganda still has limited information on ECC and its burden on children particularly from the rural communities within Uganda. Furthermore, the Ugandan health sector is still faced with challenges like shortage of human resource in the public hospitals, irregular/ineffective supervisions of health services offered at these public hospitals, limited financial resource within the national budget and a high attrition rate of the oral health care professionals in the public health sector (13). All these factors are likely to affect the provision of the requisite oral health care to most of the children with ECC residing in the rural communities.

The purpose of the current study was, therefore, to determine the prevalence and treatment needs for ECC in a rural child population in Uganda.

## Methodology

### Study Design and Study Population

This was a descriptive cross-sectional oral health survey conducted in 2016 and involved 3–5-year-old nursery school children in government and private primary schools in Nyakagyeme sub-county, Rukungiri District in South-western Uganda.

### Sampling

The study population was drawn from the 25 primary schools in Nyakagyeme Sub-county each with pre-primary/nursery section. Using the Uganda national population census results of 2014, the sub-county was estimated to have ~ <6,000 children aged 5 years and below. Stratified random sampling procedure was used to select the schools with the aim of selecting at least one school in each parish. The sub-county has 8 parishes and thus 8 strata were formed and each parish was given a code (P1–P8). The list of the schools in each parish also was generated and from the lists formed, each school in the parish was assigned a unique letter code. The schools in the parishes that had at least 50 pupils or more (18 schools), whose ages were 3–5 years age, were eligible for selection. Ballots with the number assigned to each of the selected schools in each parish were made, folded and mixed in a box. If a parish had no school with at least 50 pupils in nursery, the school with the highest number of pupils was then selected for inclusion in the study. Thereafter, the principal investigator (PI) then randomly selected one school from each parish by picking a ballot from the box. A total of 8 schools were thus selected from the sub-county and these schools has a total number of 619 children eligible for participation in the study. The participating schools' register was then used as the sampling frame to select the study population, provided they met the inclusion criteria, which included being in good general health and whose parents provided written informed consent for their participation in the study. The children were then examined for dental caries and the dental treatment needed as prescribed in the study.

The study population had been calculated with the assumption that, the population of children below 6 years in the sub-county was below 10,000 and the prevalence of caries among them to average 50%, a 95% confidence level being considered and 5% degree of accuracy. This gave the number of the participants as 384. However, a total of 432 children in the sub-county aged 3 to 5 years (230 males and 202 females; mean age 4.1, *SD* = 0.8) met the inclusion criteria and were examined during the survey.

### Data Collection

Prior to collection of data, Ethical clearance was obtained from Kenyatta National Hospital-University of Nairobi Ethics and Research Committee (Ref: P460/06/2016) and the School of Health Sciences Institution Review Board and Ethics Committee, Makerere University, Kampala, Uganda (SHSREC REF: 2016-036). Permission was also obtained from the Rukungiri District Education Officer (DEO) and individual participating schools, for children to participate in the study. Before the commencement of the study, the recording clerk was trained and pretested in the data collection exercise. The principle investigator (PI) was also calibrated by one of the supervisors under field conditions on detection of dental caries and dental treatment needs using 3–5-year-old children and who were not part of the study population. The process was repeated again in the field. The mean Cohen kappa score for the inter-examiner consistency were 0.90 and 0.95 (*N* = 15) for dental caries and dental treatment needs, respectively. The PI also regularly carried out intra-examiner consistency during the collection of data stage, and the mean Cohen Kappa scores were 1.00 and 0.98 (*N* = 43) for dental caries and dental treatment needs, respectively.

During the examination phase, the PI used visual and tactile methods to examine participants for dental caries. A sterile mouth mirror and WHO dental probe were used to carry out the procedure under natural light. An assessment Form (modified World Health Organization (WHO) Oral Health Assessment Form for children, 2013) (14), was used to record the findings of the clinical examination. The children's age and gender were also recorded. In carrying out the examination, individual teeth were first isolated, wiped clean and dried with sterile gauze before the detection for dental caries was undertaken. The data obtained was also used to calculate the “**dmft**” in order to get a record of the dental caries experience of the participants (14). Incipient lesions were diagnosed when a tooth had a whitish chalky area/spot on the cervical areas and occlusal surfaces with no signs of structural breakdown. A tooth was recorded as having caries whenever enamel and/or dentin breakdown was observed or detected by the WHO probe. A tooth was recorded to be missing due to caries if a history of early extraction due to caries could be confirmed by the teacher, parent and/or guardian on further inquiry or explained by the decay pattern in the contra-lateral quadrant(s) in the child. Radiographs were not used to detect caries.

The normative dental treatment needs for each participant were also assessed and recorded using the intervention urgency criteria (modified) as recommended by the WHO (14). This was made through an assessment of what treatment was needed for each child using a modified Intervention Urgency criterion from that described by the WHO. For this study, the study participants were assessed on variables that they could not ascribe a treatment need to, given their age and lack of knowledge regarding severity of ECC. Therefore, only a normative needs assessment was possible as such data would be readily available and easy to collate (15). The assistant also recorded the treatment needs for each child depending on the findings made by the PI. The PI would then cross-check with the recorded findings at the end of each session before the children were dismissed to make sure they had been coded correctly.

### Data Analysis

The Data gathered in the study were analyzed using SPSS version 23.0 to determine the descriptive and inferential statistical characteristics. Mann Whitney and Kruskal-Wallis tests were used to compare and relate the variables, with the confidence interval was set at 95%.

## Results

### Socio-Demographic Characteristics

A total of 432 children composed of, 118 (27%), 145 (33.6%), and 169 (39.1%) aged 3, 4, and 5 years, respectively, participated in the study, with 230 (53.2%) being male and 202 (46.8%) female participants. The participants had a mean age of 4.1 (*SD* = 0.8) years and a mode of 5 years. The mean age of the male participants (4.10, *SD* = 0.78) was slightly higher than that of the female participants (4.00, *SD* = 0.82) but this difference was not statistically significant [*t*_(430)_ = 0.0810, *p* = 0.418].

### Prevalence of ECC/Dental Caries Experience

Out of the 432 participants, 209 of them had frank caries, 93 had teeth with incipient lesions and 13 had missing teeth due to dental caries and only 3 participants had at least one filled tooth. Only 35 (8.1%) participants were found to have missing teeth that were not due to caries, but rather as a result of a traditional practice called “*Ebiino*,” a form of Infant Oral Mutilation (IOM) involving the primary canines in both arches. The presence of decay was found to vary in decreasing order as follows; primary second molars in both arches and primary first upper incisors (85, 75, 51, 61, 55, 65) as the most affected teeth (0.8–1.1%), and the lower incisors being the least affected by decay.

The overall prevalence of ECC in the children who participated in this study was 48.6%, with the male participants having higher prevalence of dental caries (26.1%) against the female participants' figure of 22.5%. In accordance to the age of the participants, the prevalence ranged from 11.6% (50), 18.5% (80), and 18.5% (80) for the 3-, 4-, and 5-year olds, respectively.

The mean “**dmft**” for the participants was 2.04 (*SD* = 3.01), with the mean decayed component (dt) of 1.97 (*SD* = 2.89), and the mean “**dmft**” of 2.19 (*SD* = 3.00) and 1.87 (*SD* = 3.02) for male and female participants, respectively. The difference between these means was not statistically significant (Mann–Whitney test *z* = 1.331, *p* = 0.183). The mean “**dmft**” for the 3-, 4-, and 5-year old children was, 1.79 (*SD* = 2.87), 2.37 (*SD* = 3.19), and 1.91 (*SD* = 2.93), respectively, but the difference in the mean “**dmft**” among the age groups was also not statistically significant (chi-squared = 3.250, *p* = 0.1969). “Decay” contributed the most to the dental caries experience (88.6%) and “filled” was the least (0.09%) depicting a very high unmet-treatment need among the participants (Tables 1, 2).

**Table 1:** The distribution of the dental caries experience among the study participants according to their gender and age.

	**dt**	**mt**	**ft**	**dmft**
	**Mean** **±** **SD**	**Mean** **±** **SD**	**Mean** **±** **SD**	**Mean** **±** **SD**
**Overall**	1.97 ± 2.89	0.06 ± 0.38	0.002 ± 0.04	2.04 ± 3.01
**Gender**				
Male	2.10 ± 2.87	0.073 ± 0.40	0.004 ± 0.065	2.19 ± 3.00
Female	1.81 ± 2.92	0.04 ± 0.35	0 ± 0	1.87 ± 3.02
Mann-Whitney test *z* = 1.331, *p* = 0.183				
**Age**				
3 years	1.79 ± 2.87	0 ± 0	0 ± 0	1.79 ± 2.87
4 years	2.26 ± 3.05	0.09 ± 0.49	0.006 ± 0.08	2.37 ± 3.19
5 years	1.82 ± 2.76	0.08 ± 0.40	0 ± 0	1.91 ± 2.93
chi-squared = 3.250, *p* = 0.1969				
chi-squared with ties = 3.771, *p* = 0.1518				

**Table 2:** Distribution of the “**dmft**” components among the study participants.

**Characteristics**	***n***	**dt**	**mt**	**ft**
dmft	1,006	881 (87.57%)	124 (12.33%)	1 (0.09%)
Overall	8,640	881 (10.20%)	124 (1.44%)	1 (0.01%)

*dt, decayed teeth; mt, missing teeth; ft, filled teeth. n = 8,640 (total number of teeth examined)*.

### Dental Treatment Needs

**Figure 1:**
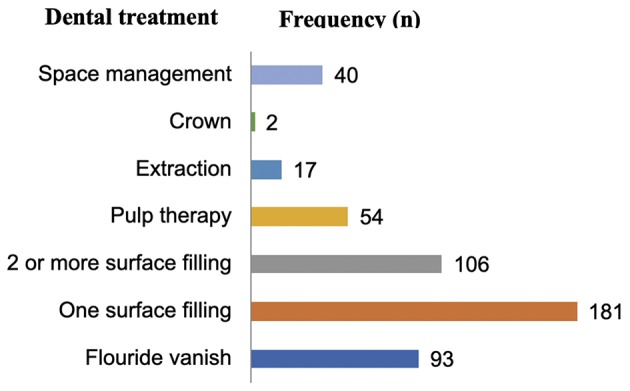
The dental treatment needs among the study participants.

The PI evaluated the dental treatment needs for the participants through the assessment of the status of individual teeth and the treatment needs. Decayed teeth contributed to the largest need for treatment, with 181 of the participants having at least a tooth that needed a one surface filling, 106 having at least one tooth in need of a 2 or more surface filling and only 2 children needed a stainless-steel crown as the primary treatment because they had some primary molars that were hypoplastic, in addition to the 54 participants who had teeth that would require pulp therapy prior to placement of stainless-steel crown (Figure 1). A total of 93 children had incipient lesions that would benefit from fluoride varnish application. However, given the high prevalence of decay seen, all children would benefit from this form of treatment. Space management was required in 40 children as a result of missing teeth majorly contributed by teeth missing due to “*Ebiino*.”

## Discussion

### Prevalence of ECC

The current study on the prevalence and treatment needs of ECC was the first of its kind to be undertaken in Nyakagyeme sub-county, Rukungiri District and the greater south western part of Uganda. Previous studies in Uganda on ECC (11, 12) were conducted in the urban and peri-urban areas of Uganda, and focused mainly on determining the prevalence of ECC and the dental caries experience among children aged 3-, 4-, and 5-years old children. Only one study (16) reported on the dental treatment needs for children aged 5–7 years from a rural sub-county in central Uganda.

The prevalence of ECC of 48.6% in this study was lower than that reported by Kiwanuka et al. (11) for central Kampala and Nakawa division in Uganda of 56 and 64%, respectively in the same age group. Comparatively, the prevalence rate of ECC reported in a Sudanese study by Awooda et al. (17) in 2013 was also lower at 17.0%, and was thought to have been probably due to the criteria used to define and diagnose the ECC in that study. However, the current prevalence rate of ECC was higher than that reported by Birungi et al. (10) of 38–41% among 5-year-old children in Mbale District, Uganda. In the current study, the male participants had a higher prevalence than their female counterparts, with the dental caries experience increasing with the increase in age of the study participants. This difference in the dental caries experience between the male and female, and among the 3-, 4- and 5-year age groups, however, was not statistically significant (*p* = 0.183) and (*p* = 0.1969), respectively. The prevalence rates observed in this study were almost similar to those reported by studies conducted in Kenya (59 and 46.3%) (18, 19), Tanzania (42.5 and 59.1%) (20), India (45%) (21), Hong Kong (49%) (22), and Beijing (20–65%) (23). A look at the outcome of similar studies on the prevalence of ECC in East Africa in the recent past and how they compare to the current study is illustrated in Table 3.

**Table 3:** Comparison of the dental caries experience in the present study to similar studies done previously within the East African Region.

**Region**	**Sample**		**Dental caries**		**References**
			**Rate %**	**dmft**	
Nyakagyeme sub-county, Rukungiri District, Uganda	*n* = 432		48.6	2.04	Present study
	Gender	Female	22.5	2.19	
		Male	26.1	1.87	
	Age	3	11.6	1.79	
		4	18.5	2.37	
		5	18.5	1.91	
Kampala Central and Nakawa Division, Uganda	*n* = 586		56 and 64	–	(11)
	Age	3	45	1.7	
		4	59	2.4	
		5	65	3.1	
Mbale District, Uganda	*n* = 417				(10)
	Age	5	38 – 41	1.5–1.7	
Kiambaa Division, Kiambu County, Kenya	*n* = 336		59.9	2.46	(10)
	Gender	Female	54.9	2.29	
		Male	64.5	2.63	
	Age	3	47	1.35	
		4	55	2.31	
		5	63	2.61	
Moshi Municipality, Tanzania	*n* = 372		30.1	0.96	(24)
	Age	3	–	0.43	
		4	–	1.01	
		5	–	1.23	

The probable causative and/or risk factors of ECC in the children who participated in the current study need to be explored in order to understand better the unique oral health challenges faced by this population. Furthermore, caution needs to be exercised when interpreting the results of this study and making comparisons with results from previous studies within Uganda and other regions. This is because, at times, various age ranges for children aged below 6 years may be considered like how the Kenya National Oral health Survey and Birungi et al. studied only 5-year olds (10, 19). The diagnostic criterion for ECC can also vary among researchers, for example Awooda et al. (17), in their study only considered a child to have ECC when their maxillary primary incisors had dental caries and this contributed to the lower prevalence rate of ECC reported in that study. This in a way makes it arduous to comprehensively make comparisons of the prevalence rates of ECC within the different regions of the world with the changing times. In this study, the most affected teeth were the primary molars in both the mandibular and maxillary arches as well as the maxillary incisors, closely followed by the canines with the lower incisors being the least affected by ECC. This pattern of ECC has been observed in many other studies (2, 3, 5, 8, 9) and is thought to be pattern followed in children affected with ECC.

The decay component contributed the most to the “**dmft**” index while filled had the least contribution. Studies by Njoroge et al. (18) and Rwakatema and Nganga (24) also reported a very high percentage contributed by the decay component to the caries experience. Factors that may be contributory to this finding include; limited oral health service provision, shortage of dental health professionals, lack of awareness, and insufficient funding for oral health related treatment within the region and country at large.

### Dental Treatment Needs

The present survey also documented the normative dental treatment needs during each child's dental assessment. It was noted that, a treatment needs assessment that evaluates all the four categories of needs as described by Bradshaw (15, 25) could provide a comprehensive evaluation based on what a researcher seeks to investigate. Given that the prevalence of ECC was high and the decay component contributed to most of the dental caries experience (mean **dt** = 1.97), it was indicative that the participants had an unmet dental treatment need. This was reflected in the high number (66.4%) of children who needed at least one surface or 2-surface filling as a form of treatment. At least 12.5% of the children needed pulp therapy; probably this figure could have increased if the assessment involved radiographic examination which would have influenced the decision made regarding the proposed treatment of certain lesions.

The dental treatment needs reported in this study were relatively similar to the dental treatment needs reported for pre-school children from Narmada district, in Gujurat, India that were reported in 2015 by Dixit et al. (26). A previous Ugandan study on dental treatment needs among 5–7-year-old children had noted that 52.7% of the children needed fillings or a dental extraction. Among those that needed fillings, 36.4% needed one or more surface fillings (16).

The reasons for the high number of untreated dental decay in the current study could be related to, lack of knowledge regarding oral health, shortage of oral health professionals and related services at the respective health centers and the district hospital as earlier noted. Dental treatment is reported to be very expensive (2, 27) and given that the people in Nyakagyeme sub-county are rural peasant farmers (28) and oral health care in the Ugandan health system faces lots of challenges (13), it is highly unlikely that this population would afford the cost of treatment from private facilities. Thus, for such a population, preventive measures could play a key role in ensuring that the suffering these children experience when affected by dental caries and other oral health related problems is minimized, but again the shortage and/or lack of human resource would pose a significant challenge to successfully implement these measures.

One of the local oral health issue noted in the study population was that of “*Ebiino*,” which is a traumatic and heinous traditional practice that should not be done to any child in the world. It is possible that through the formulation and effective implementation of appropriate national oral health policies and programmes on dental health education and prevention, control of most these common oral condition in these children can be achieved country-wide. These include, provision of adequate and skilled oral health professionals to provide advice and oral health care, adherence to proper oral hygiene instructions, appropriate and effective tooth brushing using fluoridated toothpaste and brushing as soon as the primary teeth erupt (6, 29).

## Conclusion

The overall prevalence of ECC in the study population of 48.6% is high with the “decay” component (**dt**) contributing the most (88.6%) to the dental caries experience. This has represented a high level of unmet dental treatment need in the study population besides the practice of “*Ebiino*” that could result in possible future orthodontic treatment needs.

There is therefore, a need for intervention programmes like oral health education and outreach programmes to support this community in dealing with dental caries and the traditional practice of “*Ebiino*.”

## Data Availability

The datasets for this manuscript are not publicly available because the data set was handed over to the University of Nairobi Library for Storage and archiving. Requests to access the datasets should be directed to musakulu@gmail.com.

## Ethics Statement

The study was carried out in accordance with the recommendation of the International Ethical Guidelines for Epidemiological Studies of the World Health organization and approved by Kenyatta National Hospital-University of Nairobi Ethics and Research committee (Ref: P460/06/2016) and the School of Health Sciences Institutional Review Board and Ethics committee, Makerere University Kampala, Uganda (SHSREC REF: 2016-036) with written informed consent obtained from the parents and/or guardians of all the subjects. All parents of the participants gave written informed consent in accordance with the declaration of Helsinki and the protocol approved by the School of Health Sciences Institutional Review Board and Ethics committee, Makerere, University, Kampala, Uganda. All the participants accents to be examined and only health children without any underlying medical illness or condition were included in this study.

## Author Contributions

NM designed the current study as part of his Master's Dissertation and, in collaboration with AK and IO, wrote the research protocol. NM with the assistance of AK designed the aims of the study and NM clinically examined the study participants and collected data under the supervision of IO while collaborating with AK. NM and AK analyzed the Data and NM wrote the initial draft of the manuscript. All authors participated in the interpretation of the data, made corrections to the manuscript, and approved the submitted version of the manuscript.

### Conflict of Interest Statement

The authors declare that the research was conducted in the absence of any commercial or financial relationships that could be construed as a potential conflict of interest.

## References

[1] Petersen PE Bourgeois D Ogawa H Estupinan-Day S Ndiaye C. The global burden of oral diseases and risks to oral health. Bull World Health Organ. (2005) 83:661–9. 16211157 PMC2626328

[2] Kemoli AM. Global disparity in childhood dental caries: is there a remedy? East Afr Med J. (2013) 90:130–6. 26866097

[3] Zafar S Harnekar S Siddiqi A Early childhood caries: etiology, clinical considerations, consequences and management. Int Dent Sa. (2009) 11:24–36.

[4] Borutta A Wagner M Kneist S Early Childhood caries: a multi-factorial disease. OHDMBSC. (2010) 9:32–8.

[5] Colak H Dulgergil CT Dalli M Hamidi MM. Early childhood caries update: a review of causes, diagnoses, and treatments. J Nat Sci Biol Med. (2013) 4:29–38. 10.4103/0976-9668.107257 23633832 PMC3633299

[6] Dean JA Jones J Walker Vinson LQA McDonald and Avery's Dentistry for the Child and Adolescent - (Tenth Edition). ScienceDirect: Elsevier Inc (2016). Available online at: https://www.sciencedirect.com/science/book/9780323287456

[7] Alazmah A. Early childhood caries. J Contemp Dent Pract. (2017) 18:1–6. 10.5005/jp-journals-10024-2116 28816199

[8] Kawashita Y Kitamura M Saito T. Early childhood caries. Int J Dent. (2011) 2011:725320. 10.1155/2011/725320 22007218 PMC3191784

[9] Fung MHT Wong M Lo ECM Chu CH Early childhood caries: a literature review. Oral Hyg Health. (2013) 1:1–7. 10.4172/2332-0702.1000107

[10] Birungi N Fadnes LT Okullo I Kasangaki A Nankabirwa V Ndeezi G. Effect of breastfeeding promotion on early childhood caries and breastfeeding duration among 5 year old children in eastern uganda: a cluster randomized trial. PLoS ONE. (2015) 10:e0125352. 10.1371/journal.pone.0125352 25938681 PMC4418833

[11] Kiwanuka SN Astrom AN Trovik TA. Dental caries experience and its relationship to social and behavioural factors among 3–5-year-old children in Uganda. Int J Paediatr Dent. (2004) 14:336–46. 10.1111/j.1365-263X.2004.00570.x 15330999

[12] Masumo R Bardsen A Mashoto K Astrom AN. Prevalence and socio-behavioral influence of early childhood caries, ECC, and feeding habits among 6–36 months old children in Uganda and Tanzania. BMC Oral Health. (2012) 12:24. 10.1186/1472-6831-12-24 22834770 PMC3434064

[13] Muhirwe LB. Oral health in Uganda: the need for a change in focus. Int Dent J. (2006) 56:3–6. 10.1111/j.1875-595X.2006.tb00067.x 16515006

[14] WHO Oral Health Surveys Basic Methods. 2013.

[15] Watson MC Normative needs assessment: Is this an appropriate way in which to meet the new public health agenda? Inter J Health Promo Edu. (2004) 40:4–8.

[16] Nalweyiso N Busingye J Whitworth J Robinson PG. Dental treatment needs of children in a rural subcounty of Uganda. Int J Paediatr Dent. (2004) 14:27–33. 10.1111/j.1365-263X.2004.00514.x 14706025

[17] Awooda EM Saed SM Eibasir IE Caries prevalence among 3–5 year old children in Khartoum state, Sudan. Innov J Med Health Sci. (2013) 3:42–4.

[18] Njoroge NW Kemoli AM Gatheche LW. Prevalence and pattern of early childhood caries among 3–5 year olds in Kiambaa, Kenya. East Afr Med J. (2010) 87:134–7. 10.4314/eamj.v87i3.62199 23057310

[19] Ministry Of Health Republic of Kenya Kenya National Oral Health Survey Report (2015).

[20] Maro D Kahabuka FK Prevalence of ECC among 2–6 years old underprivileged and privileged children in Dar es salam. Tanzania Dental J. (2007) 14:53–8. 10.4314/tdj.v14i2.37571

[21] Gupta D Momin RK Mathur A Srinivas KT Jain A Dommaraju N. Dental Caries and Their Treatment Needs in 3–5 Year Old Preschool Children in a Rural District of India. N Am J Med Sci. (2015) 7:143–50. 10.4103/1947-2714.156010 25973401 PMC4426517

[22] Chu C-H Ho P-L Lo EC. Oral health status and behaviours of preschool children in Hong Kong. BMC Public Health. (2012) 12:767. 10.1186/1471-2458-12-767 22966820 PMC3490858

[23] Fan C Wang W Xu T Zheng S. Risk factors of early childhood caries among children in Beijing: a case-control study. BMC Oral Health. (2016) 16:98. 10.1186/s12903-016-0289-6 27639848 PMC5027078

[24] Rwakatema DS Ng'ang'a PM. Early childhood caries in Moshi, Tanzania. East Afr Med J. (2010) 87:304–10. 23451550

[25] Pekiner F Gumru B Borahan MO Aytugar E. Evaluation of demands and needs for dental care in a sample of the Turkish population. Eur J Dent. (2010) 4:143–9. 20396444 PMC2853821

[26] Dixit A Aruna DS Sachdev V Sharma A Prevalence of dental caries and treatment needs among 3–5 year old preschool children in Narmada, Gujarat. IOSR J. Dental Med Sci. (2015) 14:97–101.

[27] Petersen PE. The World Oral Health Report 2003: continuous improvement of oral health in the 21st century–the approach of the WHO Global Oral Health Programme. Community Dent Oral Epidemiol. (2003) 31 (Suppl. 1):3–23. 10.1046/j.2003.com122.x 15015736

[28] Rukungiri District Higher Local Government Statistical Abstract (2009).

[29] Davies RM Davies GM Ellwood RP Kay EJ. Prevention. Part 4: what advice should be given to patients? Br Dent J. (2003) 195:135–41. 10.1038/sj.bdj.4810396 12907975

